# Supplementary material for: Experimental evidence of quantum radiation reaction in aligned crystals
Source: Nat Commun. 2018 Feb 23;9:795. doi: 10.1038/s41467-018-03165-4 (PMC5824952; doi:10.1038/s41467-018-03165-4)
Supplement: Supplementary file 1 — Supplementary Information [file 41467_2018_3165_MOESM1_ESM.pdf]

Experimental Evidence of Quantum Radiation Reaction in Aligned Crystals

Tobias N. Wistisen,[1*] Antonino Di Piazza,[2] Helge V. Knudsen,[1] Ulrik I. Uggerhøj[1]

[1]Department of Physics and Astronomy, Aarhus University,
Ny Munkegade 120, Aarhus, 8000, Denmark
[2]Max Planck Institute for Nuclear Physics,
Saupfercheckweg 1, Heidelberg, 69117, Germany

*To whom correspondence should be addressed; E-mail: tobiasnw@phys.au.dk.

January 24, 2018

Supplementary Note 1

Theoretical Models

We have considered four different theoretical models to compare with the experiment (units with $\hbar = c = 1$ are employed):

Model 1: Classical plus radiation reaction model (CRRM). In this model, we include radiation reaction classically, that is, we determine the positron trajectory via the Landau-Lifshitz (LL) equation $mdu^i/ds = qF^{ij}u_j + f^i$, where (see, for example, (*1*))

$$f^i = \frac{2q^3}{3m}\frac{\partial F^{ik}}{\partial x^l}u_k u^l - \frac{2q^4}{3m^2}F^{il}F_{kl}u^k + \frac{2q^4}{3m^2}(F_{kl}u^l)(F^{km}u_m)u^i. \tag{1}$$

Here, u^i is the positron four-velocity, s its proper-time, $q = e > 0$ and m its charge and mass respectively, and F^{ij} the electromagnetic field tensor of the crystal (see, for example, (*2*)). The crystal field has been modeled starting from from the sum of Doyle-Turner string potentials (*3*),

centered on a regular grid according to the diamond cubic crystal structure. Once the positron trajectory has been determined, the emission spectrum has been computed starting from the Liénard-Wiechert potential and following the standard procedure, as described, for example, in (*2*) and based on the expression of the differential intensity of radiation $dI/d\omega d\Omega$ per unit of emitted radiation frequency ω and solid angle Ω:

$$\frac{dI}{d\omega d\Omega} = \frac{e^2}{4\pi^2} \left| \int dt \frac{\boldsymbol{n} \times [(\boldsymbol{n} - \boldsymbol{\beta}) \times \dot{\boldsymbol{\beta}}]}{(1 - \boldsymbol{n} \cdot \boldsymbol{\beta})^2} e^{i\omega(t - \boldsymbol{n} \cdot \boldsymbol{r})} \right|^2 , \tag{2}$$

where the electron position $\boldsymbol{r}(t)$, velocity $\boldsymbol{\beta}(t) = \boldsymbol{u}(t)/u^0(t)$, and acceleration $\dot{\boldsymbol{\beta}}(t) = d\boldsymbol{\beta}(t)/dt$ are obtained from the solution of the LL equation, and where $\boldsymbol{n}$ is the direction of observation corresponding to the solid angle Ω.

Model 2: Semiclassical plus radiation reaction model (SCRRM). In this model we partially include quantum effects following an approach described, for example, in (*4*), where the term involving the derivative of the field in supplementary Eq. (1) is neglected and the remaining two are multiplied by the ratio $g(\chi(x))$ between the quantum total emitted power and the corresponding classical quantity calculated at the local value $\chi(x)$ of the quantum nonlinearity parameter, where (see, for example, (*5*)):

$$g(\chi) = \frac{1}{2\pi\sqrt{3}\chi^2} \int_0^\infty du \, \frac{u(4 + 5u + 4u^2)}{(1 + u)^4} K_{2/3} \left(\frac{2u}{3\chi} \right) , \tag{3}$$

with $K_\nu(\cdot)$ being the modified Bessel function of order ν.

The emission spectrum has then been evaluated as in the CRRM. This model phenomenologically takes into account that quantum effects reduce the total radiation yield but it does not account for the intrinsic stochasticity of the photon emission process (see, for example, (*6*)).

Model 3: Quantum plus radiation reaction model (QRRM). In this model the radiation emission is taken into account fully quantum mechanically, that is, the positron propagates classically within the crystal according to the Lorentz equation and, in a genuinely random way,

it emits photons and undergoes the corresponding recoil. Notice that this model thus takes into account the quantum diffusion already measured in synchrotron machines (*7–10*). A numerical program has been written to determine the positron dynamics and emission spectrum according to the following procedure. At each step in the time evolution of the positron trajectory the probability dW/dt of single photon emission in that time interval is originally calculated within the constant-crossed field (CCF) approximation (see, for example (*11*)):

$$\frac{dW}{dt} = \frac{\alpha}{3\pi\sqrt{3}}\frac{m^2}{\varepsilon(t)}\int_0^\infty du\,\frac{5u^2+7u+5}{(1+u)^3}K_{2/3}\left(\frac{2u}{3\chi(t)}\right), \tag{4}$$

where $\varepsilon(t)$ and $\chi(t)$ are the positron energy and the value of the quantum parameter at time t (the quantum parameter being calculated at the corresponding position of the positron) and a random number generator decides if the emission takes place or not (the time step has been chosen sufficiently small such that the resulting single-photon emission probability is much smaller than unity). In the former case, on the one hand, the photon energy is also determined by sampling another random number using the procedure shown in (*12*), such that the emitted photons are consistently distributed in accordance with the formula for radiation emission also in the CCF approximation (see, for example, (*11*)):

$$\frac{dW}{dtdu} = \frac{\alpha}{\pi\sqrt{3}}\frac{m^2}{\varepsilon(t)}\frac{1}{(1+u)^2}\left\{\frac{1+(1+u)^2}{1+u}K_{2/3}\left(\frac{2u}{3\chi(t)}\right) - \int_{2u/3\chi(t)}^\infty dz\,K_{1/3}(z)\right\}, \tag{5}$$

where the variable $u = \omega/(\varepsilon - \omega)$ is employed rather than the emitted photon energy. On the other hand, the momentum of the emitted photon is directed along the positron momentum at the instant of emission (which is an excellent approximation in the ultrarelativistic regime) and it is subtracted from the positron momentum before the trajectory solver starts out with the new initial conditions according to the Lorentz equation. As we have mentioned, the emission probabilities within the CCF approximation are employed here (like in for example (*6, 13, 14*)). However, since the parameter ξ is in some cases only comparable to unity in the experiment, the model has been improved and the straightforward kinetic approach as described, for example,

in (*11*) is insufficient. In order to see this more quantitatively, we show in supplementary figure 1 panel a) the differential probability of photon emission calculated within the CCF approximation (green curve) and the more accurate approach using the general formula for the differential intensity of radiation (see (*11*) and also (*15–17*)):

$$\begin{aligned}\frac{dI}{d\omega d\Omega} =& \frac{e^2}{4\pi^2}\left\{\frac{\varepsilon^2+\varepsilon'^2}{2\varepsilon^2}\left|\int dt \frac{\boldsymbol{n}\times[(\boldsymbol{n}-\boldsymbol{\beta})\times\dot{\boldsymbol{\beta}}]}{(1-\boldsymbol{n}\cdot\boldsymbol{\beta})^2}e^{i\omega'(t-\boldsymbol{n}\cdot\boldsymbol{r})}\right|^2\right. \\ &\left. +\frac{\omega^2 m^2}{2\varepsilon^4}\left|\int dt\frac{\boldsymbol{n}\cdot\dot{\boldsymbol{\beta}}}{(1-\boldsymbol{n}\cdot\boldsymbol{\beta})^2}e^{i\omega'(t-\boldsymbol{n}\cdot\boldsymbol{r})}\right|^2\right\},\end{aligned} \tag{6}$$

with $\varepsilon' = \varepsilon - \omega$, $\omega' = \omega\varepsilon/\varepsilon'$, and whose numerical procedure is outlined in (*16*). All the numerical parameters are then chosen by matching the experimental ones except that the thickness of the crystal is only 0.1 mm (thin crystal case). In this case, in fact, essentially at most one photon is emitted by each positron such that the single photon probability already provides a measure of the real spectrum. The figure clearly shows that under the conditions of our experiment, the CCF overestimated the yield of low-energy photons. It is not clear how this approach can be extended to a thick crystal but the problem shown in this case will to some degree persist also in the case of thick crystals, that is, when multiple photon emissions and thus radiation reaction effects come into play. The disagreement especially at low photon energies can be understood in the following way. For low energies of the emitted photons $\omega \ll \varepsilon_0$, the formation length of the emission process is given by $l_f(\omega) = 2\gamma^2/\omega$, where γ is the Lorentz factor of the positron at the instant of emission (*11*). Thus, if we denote as λ_0 the typical oscillation length of a channeled positron, we expect that the CCF approximation does not work for frequencies lower than ω_c, with $l_f(\omega_c) = a\lambda_0/2$, where a is a constant of the order of unity. In order to determine the constant a, we have used the fact that at low photon energies, where it is not valid, the CCF approximation significantly overestimates the emitted radiation yield with respect to the more general and more accurate approach of Baier

et. al. based on supplementary Eq. (6), described in (*11, 15–17*), and also used numerically in several channeling codes (*18, 19*). This result is shown in supplementary figure 1. Thus, as the simplest approach, we have modified the CCF emission probability by setting it to zero for photon frequencies below ω_c. The constant a has been fixed by requiring that the resulting total yield coincides with the total yield given by the more accurate approach by Baier et al. (*11, 15–17*) (see supplementary Eq. (6)) in the case of a thin crystal where multiple photon emissions are negligible. Indeed, we have found numerically that in this way a turns out to be approximately equal to 0.52.

Model 4: Quantum with no radiation reaction model (QnoRRM). This model is the same as the QRRM described above but whenever the positron emits a photon, its recoil energy-momentum is not subtracted from the positron. The spectrum in the QRRM approaches the spectrum of this model when the crystal becomes thin because for a thin crystal the probability of multiple photon emission becomes negligible and each positron essentially emits a single photon. Thus, the difference between this model and the QRRM shows the size of the effects of the recoil of multiple photon emission, that is, of radiation reaction.

Supplementary Note 2

Simulation of experimental setup

It is clear that the photon energy spectrum originating from the tracking procedure can not be directly compared to the theoretical spectra because the response of this setup is complicated by practical issues. For example, a positron entering the setup at the center of the detector M1 and another one entering at the border of the same detector will have a different chance of leading to a detected pair. The reason is that the pair originating from the positron hitting M1 at the border is more likely to be deflected outside M5 or M6. A similar effect takes place when considering the angle of the incoming positrons. In addition to this, multiple scattering in the converter foil,

air and detectors influence both the efficiency and the resolution. In order to deal with these issues, a code simulating the setup has been written. The beam distribution in position and angle as experimentally measured are given as input to the program simulating the setup, and then the effects of multiple Coulomb scattering between and inside the detectors and converter foil, as well as of the Bethe-Heitler pair-production are included for determining the particles' dynamics (*20*). The only non-trivial input to such a simulation of the setup, is the spectrum of the radiation emitted by the positrons in the crystal, which we have determined theoretically according to the four models described above. Finally, the simulation of the setup produces data-files of the same format as those obtained from the data acquisition in the experiment, which are then both sent through the tracking algorithm. In supplementary figure 1 panel b) we have shown how the setup resolves the case of a monochromatic photon source at various energies. It is seen how the resolution (peak width) is dependent on the photon energy and the size is dependent on the rate. These calculations are based on assuming the same photon emission probability as in bremsstrahlung from a 1 cm target but that they are all emitted with the same photon energy corresponding to the labels in supplementary figure 1. The curve with the label, rate x10, illustrates the same as with the other 10 GeV curve but with a 10 times higher rate from the target, and then the result divided by 10. The difference between the two curves thus shows that some particles are not measured due to the high rate (saturation effect).

Supplementary Note 3

Reduced chi-squared statistic

In order to make a quantitative comparison of the different models as compared to the experimental data, we have performed the reduced chi-squared test for the 3.8 mm case, for the 10.0 mm case, and for the combined case, see Supplementary Table 1. The important quantity χ^2_ν is defined as $\chi^2_\nu = \nu^{-1} \sum_i (O_i - C_i)^2/\sigma_i^2$, where ν is the number of degrees of freedom, σ_i^2 is the

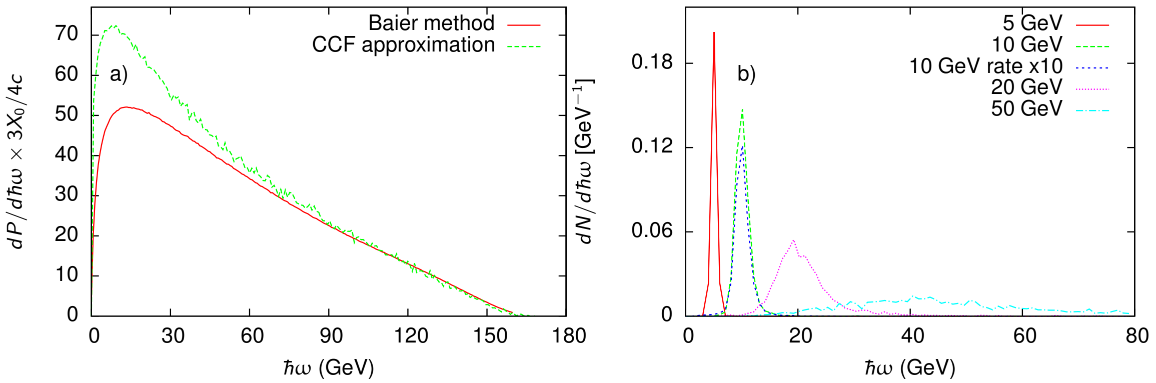

Supplementary Figure 1: The Baier method and experimental resolution. In panel a) is shown a comparison of the power spectrum for a 0.1 mm crystal using the CCF approximation and the more general approach by Baier et.al. (*11*). In panel b) we have assumed that the 1 cm target radiates with the same emission probability as if emitting bremsstrahlung, but assumed a monochromatic photon energy distribution with the energy given in the labels.

experimental error, O_i the experimental value, and C_i the calculated value. Here, $\nu = 150$ is the number of bins. A value of χ^2_ν around unity means that there is agreement between the theory and the experiment, whereas a value much larger than unity means disagreement between theory and experiment, that is, that the disagreement is not due to fluctuations."

Supplementary Table 1

Supplementary Table 1: χ^2_ν statistic for the employed theoretical models.

	QRRM	QnoRRM	SCRRM	CRRM
3.8mm	47.4	187.7	50.2	22.4
10.0mm	28.9	100.3	104.4	350.7
Comb.	38.2	144.0	77.3	186.6

Supplementary References

1. Landau, L. D. & Lifshitz, E. M. *The Classical Theory of Fields* (Elsevier, Oxford, 1975).

2. Jackson, J. D. *Classical Electrodynamics* (Wiley, New York, 1975).

3. Møller, S. P. High-energy channeling - applications in beam bending and extraction. *Nucl. Instrum. Meth. Phys. Res. A* **361**, 403 – 420 (1995).

4. Sokolov, I. V., Nees, J. A., Yanovsky, V. P., Naumova, N. M. & Mourou, G. A. Emission and its back-reaction accompanying electron motion in relativistically strong and qed-strong pulsed laser fields. *Phys. Rev. E* **81**, 036412 (2010).

5. Ritus, V. I. Quantum effects of the interaction of elementary particles with an intense electromagnetic field. *J. Sov. Laser Res.* **6**, 497 (1985).

6. Neitz, N. & Di Piazza, A. Stochasticity effects in quantum radiation reaction. *Phys. Rev. Lett.* **111**, 054802 (2013).

7. Sands, M. Observation of quantum effects in an electron synchrotron. *Il Nuovo Cimento* **15**, 599–605 (1960).

8. Gutbrod, F. Quantentheoretische behandlung der strahlungsrückwirkung relativistischer elektronen in axialsymmetrischen magnetfeldern. *Z. Phys.* **168**, 177–194 (1962).

9. Kolomenskij, A. A. & Lebedev, A. N. The theory of electron motion in cyclic accelerators in presence of radiation. *Il Nuovo Cimento* **7**, 43–60 (1958).

10. Ternov, I. M. Synchrotron radiation. *Phys. Usp.* **38**, 409 (1995).

11. Baier, V. N., Katkov, V. M. & Strakhovenko, V. M. *Electromagnetic Processes at High Energies in Oriented Single Crystals* (World Scientific, Singapore, 1998).

12. Yokoya, K. A Computer Simulation Code for the Beam-beam Interaction in Linear Colliders. *SLAC KEK-Report-85-9* (1985).

13. Blackburn, T. G., Ridgers, C. P., Kirk, J. G. & Bell, A. R. Quantum radiation reaction in laser-electron-beam collisions. *Phys. Rev. Lett.* **112**, 015001 (2014).

14. Di Piazza, A., Hatsagortsyan, K. Z. & Keitel, C. H. Quantum radiation reaction effects in multiphoton compton scattering. *Phys. Rev. Lett.* **105**, 220403 (2010).

15. Belkacem, A., Cue, N. & Kimball, J. Theory of crystal-assisted radiation and pair creation for imperfect alignment. *Phys. Lett. A* **111**, 86 – 90 (1985).

16. Wistisen, T. N. Interference effect in nonlinear compton scattering. *Phys. Rev. D* **90**, 125008 (2014).

17. Wistisen, T. N. Quantum synchrotron radiation in the case of a field with finite extension. *Phys. Rev. D* **92**, 045045 (2015).

18. Bandiera, L., Bagli, E., Guidi, V. & Tikhomirov, V. V. Radcharm++: A c++ routine to compute the electromagnetic radiation generated by relativistic charged particles in crystals and complex structures. *Nucl. Instr. Methods Phys. Res. B* **355**, 44 – 48 (2015).

19. Guidi, V., Bandiera, L. & Tikhomirov, V. V. Radiation generated by single and multiple volume reflection of ultrarelativistic electrons and positrons in bent crystals. *Phys. Rev. A* **86**, 042903 (2012).

20. Beringer, J. *et al.* Review of particle physics. *Phys. Rev. D* **86**, 010001 (2012).
